# Antimicrobial activity of NK cells to *Trypanosoma cruzi* infected human primary Keratinocytes

**DOI:** 10.1371/journal.pntd.0012255

**Published:** 2024-07-22

**Authors:** Keshia Kroh, Jessica Barton, Helena Fehling, Hanna Lotter, Beate Volkmer, Rüdiger Greinert, Mouna Mhamdi-Ghodbani, Andrea Vanegas Ramirez, Thomas Jacobs, Rosa Isela Gálvez

**Affiliations:** 1 Protozoa Immunology, Bernhard Nocht Institute for Tropical Medicine, Hamburg, Germany; 2 Molecular Infection Immunology, Bernhard Nocht Institute for Tropical Medicine, Hamburg, Germany; 3 Skin Cancer Center, Division of Molecular Cell Biology, Elbe Klinikum Buxtehude, Buxtehude, Germany; 4 Department of Dermatology, Bundeswehr Hospital Hamburg & Bernhard Nocht Institute for Tropical Medicine, Hamburg, Germany; The Ohio State University, UNITED STATES OF AMERICA

## Abstract

Infection with the protozoan parasite *Trypanosoma cruzi* is causative for Chagas disease, which is a highly neglected tropical disease prevalent in Latin America. Humans are primary infected through vectorial transmission by blood-sucking triatomine bugs. The parasite enters the human host through mucous membranes or small skin lesions. Since keratinocytes are the predominant cell type in the epidermis, they play a critical role in detecting disruptions in homeostasis and aiding in pathogen elimination by the immune system in the human skin as alternative antigen-presenting cells. Interestingly, keratinocytes also act as a reservoir for *T. cruzi*, as the skin has been identified as a major site of persistent infection in mice with chronic Chagas disease. Moreover, there are reports of the emergence of *T. cruzi* amastigote nests in the skin of immunocompromised individuals who are experiencing reactivation of Chagas disease. This observation implies that the skin may serve as a site for persistent parasite presence during chronic human infection too and underscores the significance of investigating the interactions between *T. cruzi* and skin cells. Consequently, the primary objective of this study was to establish and characterize the infection kinetics in human primary epidermal keratinocytes (hPEK). Our investigation focused on surface molecules that either facilitated or hindered the activation of natural killer (NK) cells, which play a crucial role in controlling the infection. To simulate the *in vivo* situation in humans, an autologous co-culture model was developed to examine the interactions between *T. cruzi* infected keratinocytes and NK cells. We evaluated the degranulation, cytokine production, and cytotoxicity of NK cells in response to the infected keratinocytes. We observed a strong activation of NK cells by infected keratinocytes, despite minimal alterations in the expression of activating or inhibitory ligands on NK cell receptors. However, stimulation with recombinant interferon-gamma (IFN-γ), a cytokine known to be present in significant quantities during chronic *T. cruzi* infections in the host, resulted in a substantial upregulation of these ligands on primary keratinocytes. Overall, our findings suggest the crucial role of NK cells in controlling acute *T. cruzi* infection in the upper layer of the skin and shed light on keratinocytes as potential initial targets of infection.

## Author summary

In our study, we explore how *T. cruzi*, the parasite causing Chagas disease, interacts with human skin cells, particularly keratinocytes. Chagas disease is a major issue in Latin America, making it crucial to understand how the parasite infects skin cells for treatment development.

We discovered that *T. cruzi* can infect keratinocytes, likely among the first cells targeted after an insect bite. Our research emphasizes the role of natural killer (NK) cells in responding to this infection, activating and producing molecules that can directly kill the parasite.

Our findings illuminate the skin’s immune response in controlling *T. cruzi* infection early on. Stimulation with interferon-gamma enhances keratinocytes’ ability to alert the immune system to the parasite’s presence.

Moreover, NK cells not only kill infected cells but also communicate with other immune cells to combat the infection. Understanding these interactions could improve Chagas disease management.

Overall, our study underscores the importance of investigating pathogen-skin cell interactions for effective treatment strategies, necessitating further research into underlying mechanisms and potential therapies.

## Introduction

In Latin America, the infection caused by *Trypanosoma cruzi* (*T. cruzi*) and the subsequent development of Chagas disease is still the foremost zoonotic illness, having a top position among the most neglected tropical diseases recognized by the World Health Organization (WHO) [1,2]. This infection initiates within the epithelial layers of the skin and is accompanied by a transient local immune response until the parasites disseminate throughout the host’s body via the lymphatics or bloodstream. Towards the resolution of the acute infection, which typically spans two months, only a small number of parasites survive, concealed within specific target organs where they can persist for a lifetime. Cutting-edge bioluminescence techniques have recently identified the skin as a potential novel reservoir in mouse models [3]. Despite the crucial role of the skin as both the point of entry and a potential reservoir for *T. cruzi* parasites, there remains a lack of comprehensive characterization regarding the impact of the infection on the predominant cell types found within the skin, as well as the subsequent local immune responses. The skin’s complexity arises from the diverse array of somatic and immune cells, as well as the distinct functional layers it comprises. Therefore, analyzing these intricate interactions becomes imperative in order to comprehend the skin as a significant immunological organ [4,5]. The control of *T. cruzi* infection relies heavily on the pivotal role of NK cells and CD8^+^ T cells, as these immune cell types emerge as the central immune mediators in combating the infection, given the obligate intracellular nature of the parasite [6–9]. This study aimed to characterize the impact of *T. cruzi* infection on human primary epidermal keratinocytes (hPEK) and assess the differential expression of immune regulatory ligands. First, to understand the kinetics of infection of hPEK, as well as the role of this cell type in orchestrating epidermal immune responses. Second, to gain insight into the specific mechanisms underlying the interplay between infected keratinocytes and the activation of NK cells. NK cells are a crucial immune cell population responsible for eliminating infected somatic cells in the skin and containing the parasite’s spread. Our findings demonstrate that *in vitro T. cruzi* infected hPEK can directly activate NK cells, resulting in increased secretion of pro-inflammatory cytokines (e.g., IFN-γ, IL-6) and cytolytic mediators (e.g., Granzyme A). Moreover, enhanced degranulation was observed through surface expression of CD107a. Utilizing the High Content Screening method developed in this study, we were able to quantify the increased cytotoxicity of NK cells. Additionally, our study shows that hPEK exhibited heightened expression of various HLA class I molecules and immune relevant ligands such as PD-L1, PD-L2 and ICAM-1 after infection and treatment with recombinant IFN-γ. Our study explores the relationship between hPEK and NK cells, uncovering the crucial role of keratinocytes in skin immunity against intracellular pathogens such as *T. cruzi*. The findings demonstrate that the direct interaction between infected keratinocytes and NK cells triggers cytokine production, cytolytic mediators, and enhanced cytotoxicity. This, in turn, may play a pivotal role in initiating a systemic robust immune response for effective pathogen defense.

## Results

### Human primary epidermal keratinocytes are an appropriate model for investigating the dynamics of *T*. *cruzi* infection within the skin

**Fig 1:**
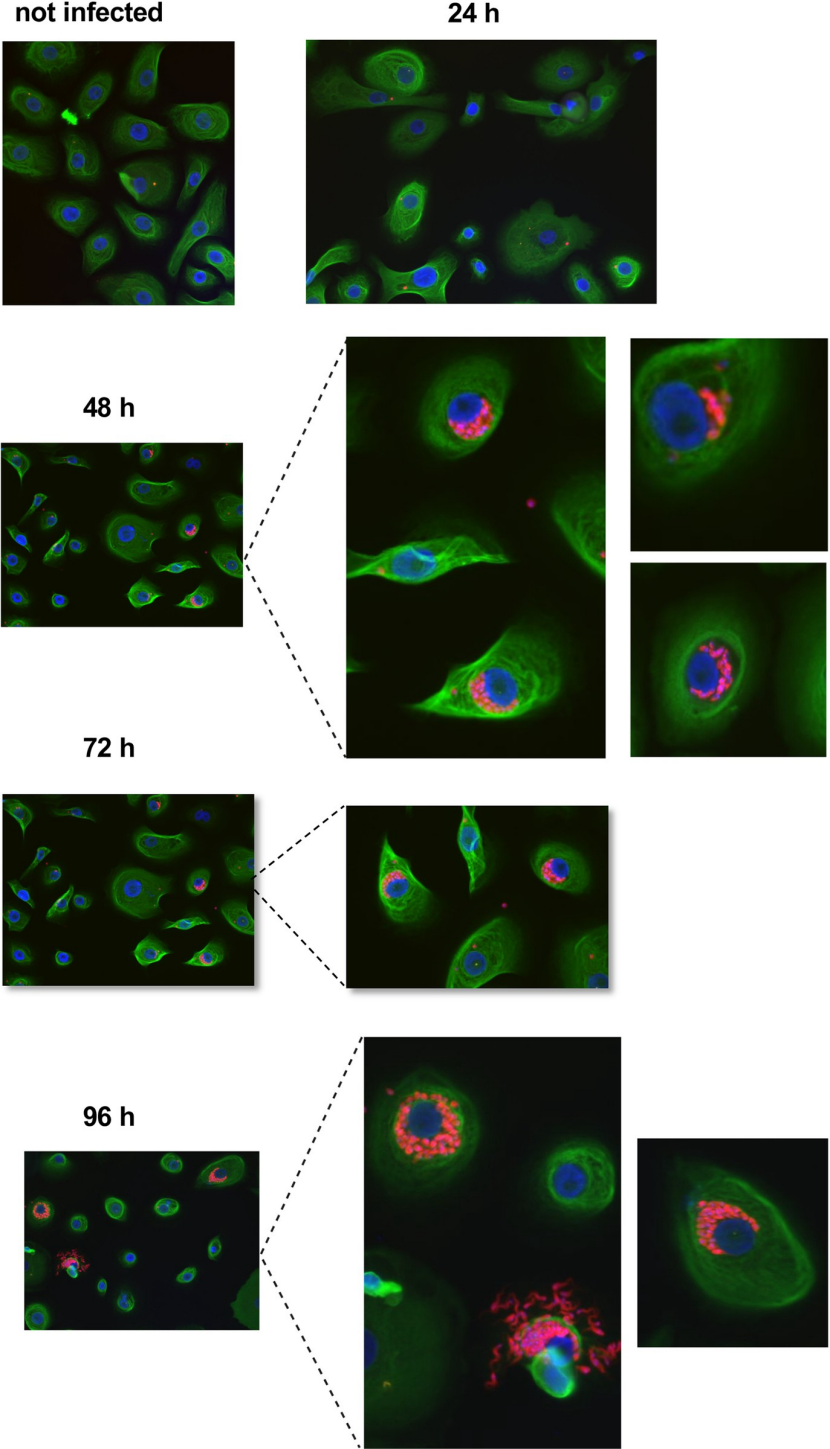
Infection of human primary keratinocytes with *T*. *cruzi* Brazil. Primary keratinocytes were infected with *T*. *cruzi* Brazil at a MOI of 3:1 for 24 h, 48 h, 72 h, and 96 h. Keratinocytes (green) and trypanosomes (red) were visualized by indirect immunofluorescence using a pan anti-cytokeratin antibody, polyclonal anti-*T*. *cruzi* serum, and DAPI. Images were obtained at 200x magnification. n.i., not infected; TcB inf., *T*. *cruzi* Brazil infected.

**Fig 2:**
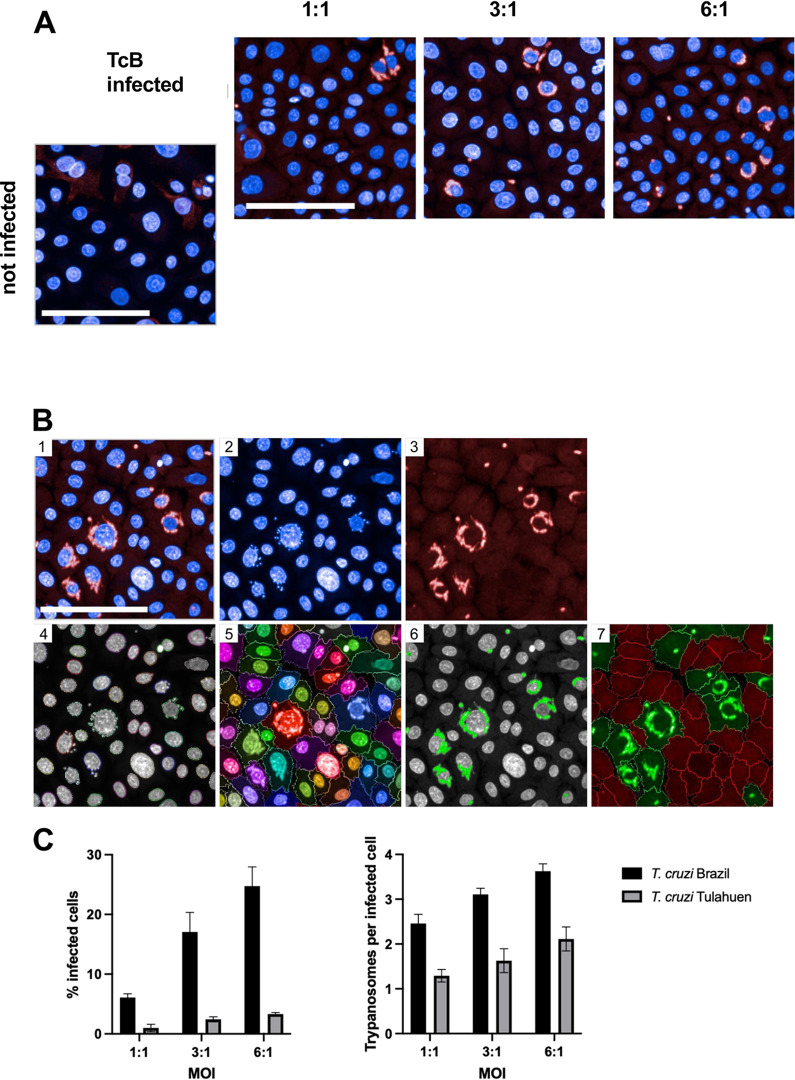
Evaluation of infection per cell using the high-content screening system Opera Phenix. Primary keratinocytes were used to determine the infection rates of *T*. *cruzi* Brazil and *T*. *cruzi* Tulahuen 72 h p.i. at an MOI of 1:1, 3:1, and 6:1. *T*. *cruzi* parasites were stained using a murine polyclonal anti-*T*. *cruzi* serum and an AF647-conjugated anti-mouse IgG secondary antibody (red). Nuclei were stained with DAPI (blue). Images were obtained using the Opera Phenix confocal imaging system. A) Representative image sections showing primary keratinocytes infected with *T*. *cruzi* Brazil, MOI of 1:1, 3:1, and 6:1. B) Image analysis sequence for one representative image (*T*. *cruzi* Brazil infected, MOI 6:1). (1) Input image, (2) nuclei staining (DAPI, 405 nm) (3) anti-*T*. *cruzi* staining (AF647, 640 nm), (4) detection of nuclei and (5) cytoplasm, (6) detection of trypanosomes, (7) selection of infected cells (green) and uninfected cells (red). C) Infection rates and average number of trypanosomes per infected cell for *T*. *cruzi* Brazil and *T*. *cruzi* Tulahuen infected primary keratinocytes. 5 wells were analyzed for each condition, and values are shown as mean ± SD. Scale 100 μm.

First, we aimed to study the kinetic of the *in vitro* infection of hPEK with two different strains of *T. cruzi*: Tulahuen and Brazil. The different infection stages were evaluated first by immunofluorescence staining 24 to 96 h post infection (p.i.) (Fig 1). While at 24 h p.i. single intracellular *T. cruzi* amastigotes were visible, the number of intracellular amastigotes per cell increased over time. The timepoint 72 h p.i. was chosen for subsequent infection experiments, as the amastigotes have filled up the cells but were not yet transforming into the blood trypomastigote stage, as seen 96 h p.i. The infection kinetics of both *T. cruzi* strains were similar, although the infection rates for *T. cruzi* Tulahuen were lower (Fig A in S1 Appendix). To confirm and automatically quantify infection rates the Opera Phenix HCS system was used and an image analysis sequence with the Harmony software was established as described in previous study by Fehling et al. [10]. To assess the infection rates at different multiplicity of infection (MOI), keratinocytes were seeded and infected with MOIs of 1:1, 3:1 and 6:1, and an infection period of 72 h. Cells were fixed and stained with DAPI (blue), and *T. cruzi* parasites were stained using polyclonal mouse anti-*T. cruzi* serum and a conjugated anti-mouse secondary antibody (red). The anti-*T. cruzi* antibody also displayed a weak, unspecific staining of cells, which was used to identify the cytoplasm. The image analysis sequence was shown to robustly detect the cells and intracellular trypanosomes (Fig 2A and 2B). *T. cruzi* Brazil showed an infection rate of 6% at a MOI of 1:1. This was increased to 17% and 25% with MOIs of 3:1 and 6:1, respectively. The infection rates of *T. cruzi* Tulahuen were much lower and were 1% at an MOI of 1:1, 2% at an MOI of 3:1, and 3% at an MOI of 6:1. The average number of trypanosomes per infected cell was also higher in *T. cruzi* Brazil infected cells and ranged from 2.5 to 3.6. while the average number of *T. cruzi* Tulahuen trypanosomes was within the range of 1.3 to 2.1 (Fig 2C). These results show that hPEK can be productively infected with *T. cruzi*, independently of the strain and allow further characterization regarding the interaction with NK cells. In the following experiments only the *T. cruzi* Brazil strain was used, since it led to more efficient infection. Additionally, our previous results in immunocompetent mice support that the *T. cruzi* Brazil strain reflects infection more accurately due to its milder fashion and persistence at low parasite burden, controlled by CD8^+^ T cells [11]. This mirrors human chronic infection, offering valuable insights for further studies.

### Autologous co-cultivation of *T*. *cruzi* infected hPEK activates NK cells and triggers cytotoxic activity

**Fig 3:**
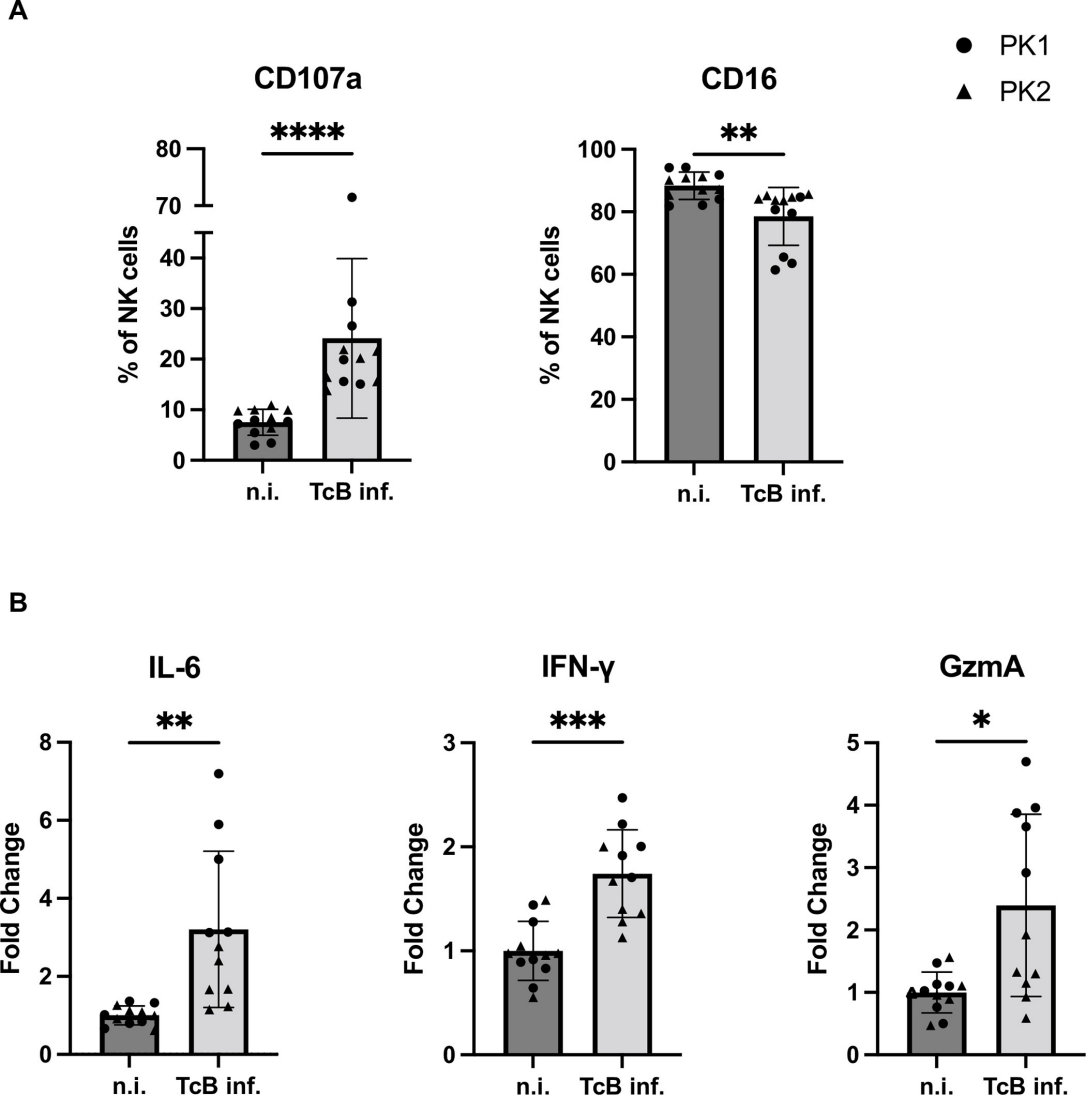
Activation of NK cells co-cultured with *T*. *cruzi* Brazil infected keratinocytes. A) Frequency of CD107a^+^ NK cells (left) and frequency of CD16^+^ NK cells (right) after an autologous co-culture with non-infected (n.i.) or *T*. *cruzi* Brazil infected (TcB) keratinocytes. Data are shown as mean ± SD. Mann-Whitney tests were performed for not normal distributed values; * p ≤ 0.05; ** p ≤ 0.01; *** p ≤ 0.001; **** p ≤ 0.0001 B) Release of cytokines and cytolytic mediators by the NK cells. The fold change was calculated in relation to the non-infected controls. n = 11–12 from 2 donors (PK1, PK2) and 2 experiments each. Data are shown as mean ± SD. Welch’s t tests were performed for normally distributed values * p ≤ 0.05; ** p ≤ 0.01; *** p ≤ 0.001; **** p ≤ 0.0001; n.i.–non-infected, TcB inf.–*T*. *cruzi* Brazil infected.

As keratinocytes may be one of the first infected cell types in the acute *T. cruzi* infection, an autologous model was employed to investigate the direct interaction between *T. cruzi* infected hPEK and NK cells from the same donor. A scheme of the co-culture experiments is shown in (Fig B in S1 Appendix). After 24 hours, supernatant of the co-culture was collected for analysis of soluble mediators and NK cells were collected for flow cytometric analysis. The gating strategy and representative dot plots are depicted in (Fig C in S1 Appendix). CD107a, which is exposed on the cell surface as a result of the degranulation of NK cells, serves as a marker for cytotoxicity [12]. The background expression of CD107a by NK cells without target cells was subtracted to evaluate the fraction of CD107a^+^ NK cells. Using this method, an average of 7.5% of NK cells co-cultured with non-infected keratinocytes, were positive for CD107a. In contrast, the proportion of CD107a^+^ NK cells in co-culture with infected keratinocytes increased to 24.1%, exhibiting a threefold increase compared to the control. In addition, CD16 was analyzed since diminished CD16 expression can occur following activation of NK cells [13]. CD16 was significantly downregulated on NK cells co-cultured with infected keratinocytes compared to non-infected keratinocytes (78.5% vs. 88.3% of CD16^+^ NK cells) (Fig 3A). A key protective mechanism employed by NK cells involves the release of cytokines and cytolytic mediators, such as lytic granules. To examine this, a multiplex bead-based immunoassay was conducted on the co-culture supernatants. The fold change in cytokine concentration was determined by comparing the mean of non-infected controls for each donor and experiment. IL-6, IFN-γ, and Granzyme A (GzmA) exhibited statistically significant differences (Fig 3B). Notably, the average fold change was higher in the supernatants of NK cells co-cultured with infected keratinocytes for all assessed cytokines and cytolytic mediators (Fig D in S1 Appendix). Overall, the observed significant increase in activation and frequency of degranulated CD3^-^CD56^+^CD107a^+^NK cells upon co-culturing with *T. cruzi* infected hPEK strongly supports the notion that infected hPEK can effectively stimulate secretion of pro-inflammatory cytokines and cytolytic mediators by NK cells.

### High-content screening assay confirms killing of infected hPEK by NK cells

**Fig 4:**
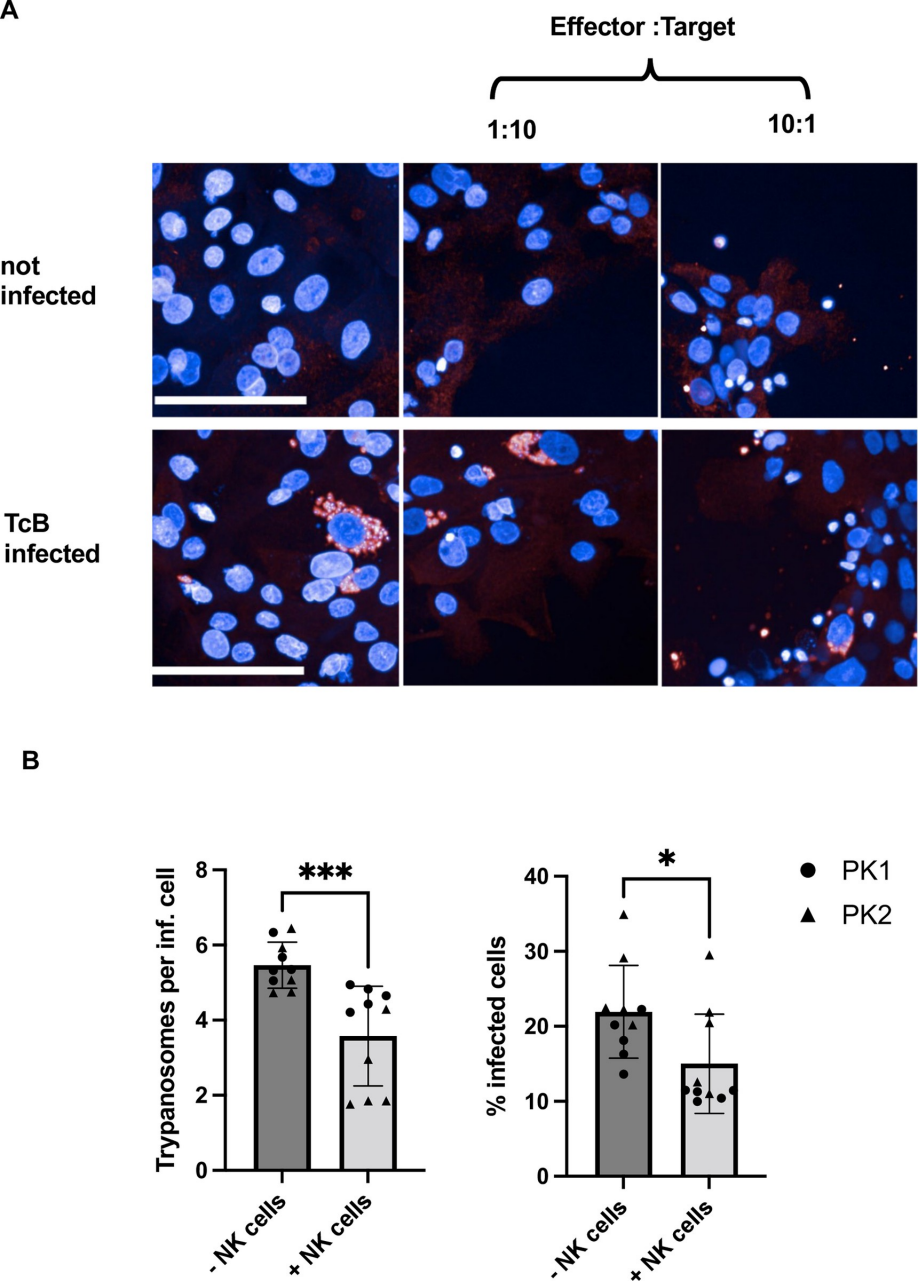
Trypanocidal effect of NK cells on *T*. *cruzi* -infected keratinocytes. A) Representative image sections of infected and non-infected keratinocytes co-cultured with NK cells at E:T ratios of 1:1 and 10:1. B) Killing effect after co-culture with NK cells is depicted as percentage of infected cells and number of trypanosomes per infected cell. This was calculated using the Opera Phenix HCS system. NK cells were added at an E:T ratio of 10:1. n = 10 from 2 donors (PK1, PK2), scale bar 100 μm. Data are shown as mean ± SD. Mann-Whitney tests were performed to calculate statistical significance. * p ≤ 0.05; *** p ≤ 0.001.

The cytotoxic effects of NK cells were assessed using the Opera Phenix high-content screening system. Confocal images of hPEK were captured after co-culturing, and a cell counting analysis sequence was employed. The evaluation included determining the percentage of infected cells and the number of trypanosomes per infected cell. Diverse effector-to-target ratios (E:T ratios), ranging from 1:10 to 10:1 were analyzed. For this, the analysis sequence was modified to exclude NK cells, which were identifiable via the much smaller nucleus compared to the nuclei of hPEKs. Image sections of infected and non-infected keratinocytes from one donor, co-cultured without NK cells or with NK cells at E:T ratios of 1:1 and 10:1 is depicted in Fig 4A. For both donors, a significant reduction in the number of trypanosomes per infected cell was observed at the highest E:T ratio of 10:1. A killing effect of NK cells on intracellular trypanosomes was observed, as both the percentage of infected cells and the number of trypanosomes per infected cell were significantly decreased with the addition of NK cells (Fig 4B).

### Upregulation of immune modulatory molecules in *T. cruzi* infected and IFN-γ stimulated human primary keratinocytes

To determine the surface expression of various molecules in hPEK infected with *T. cruzi* and stimulated with IFN-γ, flow cytometry analysis was conducted. The experimental design is shown in (Fig E in S1 Appendix). The hPEK were infected at a MOI of 3:1 or stimulated with 0.1 ng/ml and 10 ng/ml of IFN-γ respectively. IFN-γ was chosen due to its crucial role in *T. cruzi* infection in murine models [11] and also chronic Chagas patients [14]. We focused on the analysis of activating and inhibitory ligands as well as in HLA-molecules that mediate the interaction with NK cells. The gating strategy and representative dot plots are depicted in (Fig F1 and Fig F2 for activating ligands, Fig F3 for inhibitory ligands, Fig F4 for HLA-Molecules in S1 Appendix).

**Fig 5:**
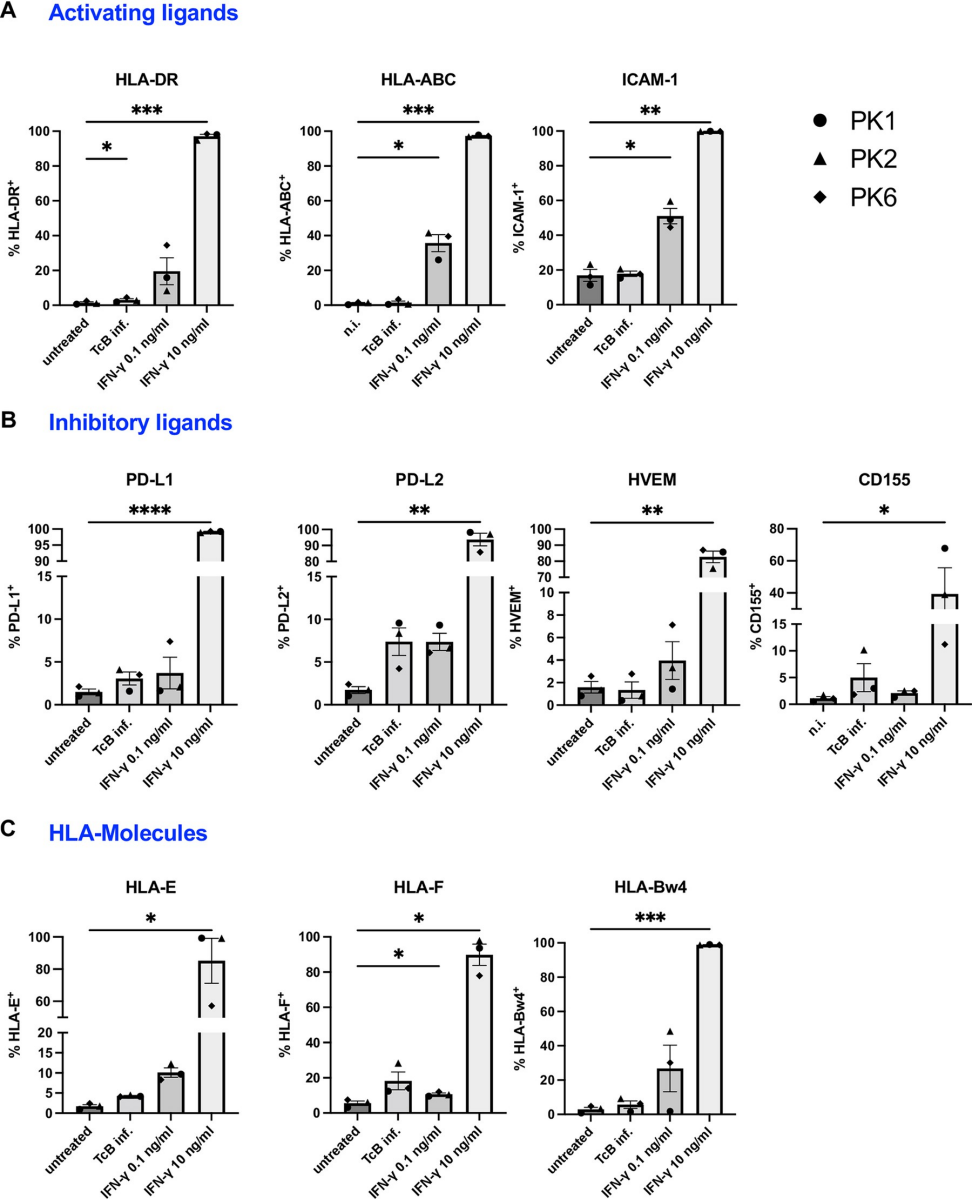
Immune modulatory molecules expression on infected and stimulated hPEK. Schematic design of the experiment in Fig E in S1 Appendix. HPEK were infected with *T*. *cruzi* Brazil at an MOI of 3:1 or stimulated with 0.1 ng/ml or 10 ng/ml of IFN-γ in duplicates. Untreated keratinocytes were used as control. 72 h after infection/stimulation, the keratinocytes were detached and stained for flow cytometric analysis. A) Frequency of keratinocytes expressing the different activating ligands, B) Inhibitory ligands, C) HLA-Molecules. Statistical differences of untreated vs. infected/stimulated cells were analyzed using RM one-way ANOVA and Holm-Sidak’s multiple comparison test for values that follow normal distribution (HLA-DR, HLA-ABC, ICAM-1, PD-L1, PD-L2, HVEM, HLA-F and HLA-Bwd) and Friedman test and Dunn’s multiple comparison test for values that do not (CD155 and HLA-E). n = 3 from three donors. Data are shown as mean ± SD. * p ≤ 0.05; ** p ≤ 0.01; *** p ≤ 0.001; **** p ≤ 0.0001. TcB inf.–*T*. *cruzi* Brazil infected.

The determination of the percentage of cells expressing the ligand was established using the negative control cell population. Consequently, the values should be interpreted as the percentage of cells expressing the target minus the background expression observed in untreated cells. Stimulation with 10 ng/ml of IFN-γ significantly increased the surface expression of nearly all analyzed targets, while the effects of *T. cruzi* infection or stimulation with 0.1 ng/ml IFN-γ were less pronounced. Specifically, the fraction of hPEK expressing the activating ligands HLA-DR and ICAM-1, and the inhibitory ligands PD-L1, PD-L2, and HVEM approached 100% after stimulation with 10 ng/ml of IFN-γ (Fig 5).

The fraction of cells expressing MIC-A/B and Galectin-9 was also higher compared to untreated controls but did not reach statistically significance (Fig F2 and Fig F3 in S1 Appendix). However, statistical significance was not achieved due to high variation between donors. With the exception of CD155, increased expression was also observed for all analyzed targets when cells were stimulated with a lower concentration of IFN-γ (0.1 ng/ml). This increase was statistically significant for HLA-ABC, HLA-DR, PD-L2, and ICAM-1. The fraction of cells positive for HLA-DR and ICAM-1 was 19.6% and 51%, respectively, while for the other targets, it ranged from 3.7% to 7.4%. The *T. cruzi* infection had a lesser impact compared to IFN-γ stimulation, although significant induction of PD-L1, PD-L2, and MIC-A/B on infected hPEK were observed. Taken together, both the upregulation of surface molecules with activating and inhibitory effects was observed. The IFN-γ stimulation induced significant upregulation of almost all analyzed molecules. This was the case for stimulation with 10 ng/ml of IFN-γ, but also a low IFN-γ concentration of 0.1 ng/ml led to a marked upregulation of most markers. The isolated *T. cruzi* infection did not have a large effect on the phenotype of primary keratinocytes with respect to the activating ligands but induced significantly inhibitory ligands.

## Discussion

Each tissue provides a unique microenvironment with a high diversity of signals affecting immune cells. These tissue-specific factors influencing innate and adaptive immune responses are not well understood. Our study aims to fill this knowledge gap by examining early stages of *T. cruzi* infection in the skin. Considering that the skin serves as the entry point for *T. cruzi*, a local immune activation orchestrates subsequent adaptive immune responses and may set the course for the outcome of infection. We specifically focus on primary skin keratinocytes, which are the predominant cell type at the site of infection. Keratinocytes constitute approximately 95% of the cell population in the epidermis, the outermost layer of the skin [15]. In acute cases of Chagas disease, patients often display a swelling in the skin, known as Romana’s sign, which results from a local inflammation at the parasite’s entry point. This swelling commonly affects the eyelid and may extend to the conjunctiva and nearby tissues. Intracellular amastigotes and immune cell infiltration can be observed in these areas. Although the Romana’s sign is a specific clinical manifestation of an acute infection with *T. cruzi*, a detailed characterization of the immunological infiltrate or a description of the early events of immune defense are lacking. Interestingly, Ward et al. have shown that *T. cruzi* is present in the skin of chronically infected mice, suggesting that keratinocytes might act as a reservoir for the parasite in individuals with chronic infections [3]. Importantly, in immune compromised patients a chronic infection is often accompanied by a reactivation with an early skin manifestation [3,16–19]. This indicates that the skin acts a reservoir and a continuous immune surveillance controls the infection.

Our data confirms that *T. cruzi* can effectively infect primary human keratinocytes. Keratinocytes are of special interest since they are likely to be the first cell type infected upon vectorial transmission. Replication within keratinocytes and recruitment of phagocytic cells as potential vehicles for dissemination will spark the initial infection. Therefore, any immune pathway capable of lowering the initial parasitic load might have a profound effect on the subsequent course of infection. NK cells have been shown to play a crucial role in controlling *T. cruzi* infection, particularly during the acute phase. Depletion of NK cells in *T. cruzi* infected mice leads to increased susceptibility, higher parasitemia, and mortality rates [9,20]. The production of IFN-γ by NK cells is considered a major protective mechanism against *T. cruzi* infection [20–22]. NK cell-mediated killing of parasites can occur through the release of granzymes, granulysin and perforin [7]. Furthermore, it is crucial to note that early IFN-γ secretion orchestrates a type I immune response that activates macrophages [23]. To address the interaction with *T. cruzi* infected hPEK an autologous model was employed, i.e., primary keratinocytes and NK cells were from the same donor. Degranulation was assessed via staining of CD107a on the surface of NK cells. CD107a is associated with the membranes of intracellular granules and, therefore, is present only on the cell surface following membrane fusion after degranulation [12]. Upon co-incubation of NK cells with infected hPEK we observed a strong increase of surface CD107a, which was not observed when NK cells were co-incubated with non-infected hPEK. Furthermore, CD16 on NK cells was significantly downregulated after co-culture with infected hPEK compared to non-infected hPEK. A loss of CD16 occurs following activation of NK cells [13]. A strong activation of NK cells is further corroborated by an increase of pro-inflammatory cytokines and cytolytic mediators in the supernatants of co-cultures. Taken together, our data provide clear evidence that NK cells are able to sense and react to *T. cruzi* infected keratinocytes. The release of granzymes, granulysin, and perforin are capable to mediate parasite killing [24], and the secretion of IFN-γ might promote a protective type I immune response and enhances the killing activity of macrophages [25,26]. To assess whether indeed NK cells exert a direct cytotoxic effect to infected hPEK the high-throughput confocal imaging system Opera Phenix was used. This analysis revealed a reduction in the total number of cells, the infection rate, and the number of *T. cruzi* parasites per infected cell with increasing E:T ratios after co-incubation with NK cells.

A reduction in the number of infected cells might result from killing by the release of cytolytic mediators. However, it is possible that direct killing is not the primary effector mechanism employed by NK cells in *T. cruzi* infections. Prajeeth et al. showed that the control of Leishmania infections by NK cells was mediated by cytokine secretion, but not by cytotoxicity [27]. In this study it was demonstrated that NK cells were not able to kill infected macrophages directly, but cytokines released by NK cells increased the antimicrobial activity of infected macrophages, such as the production of nitric oxide. Similarly, Lieke et al. showed that NK cells exhibited a trypanocidal effect on *T. cruzi* infected fibroblasts, which was mediated by soluble factors and by the production of nitric oxide.

Keratinocytes are known for their diverse immunological functions, including cytokine production, antigen presentation, and activation in response to pathogen invasion, cell damage, or a pro-inflammatory environment [28]. To further understand their role in *T. cruzi* infection, we examined the induction of immune regulatory surface molecules known to be important for the interaction with T cells and NK cells. First, we investigated whether human primary keratinocytes undergo phenotypical changes upon infection with *T. cruzi*. In addition, keratinocytes were stimulated with IFN-γ, since this cytokine is produced by CD8^+^ T cells and NK cells in *T. cruzi* infection. It has been shown to have a protective role in acute infection but also remained elevated during the chronic stage [11,21,29]. *T. cruzi* infection alone did not have a large effect on the phenotype of primary keratinocytes. This is surprising, since *T. cruzi* is able to trigger PRRs, and keratinocytes readily react to PRR stimulation with a pro-inflammatory immune response [28,30]. The results seen here would support the hypothesis that *T. cruzi* can enter the host relatively unnoticed. In contrast, IFN-γ stimulation induced significant upregulation of almost all analyzed surface molecules. This was the case for stimulation with 10 ng/ml of IFN-γ, but also low IFN-γ concentrations of 0.1 ng/ml led to a marked upregulation of most markers. This concentration falls within the range observed in the supernatants of NK cell co-culture experiments in reaction to *T. cruzi* infected keratinocytes (0.06–0.9 ng/ml). This information is presented as a fold change in Fig 3. It is important to note, however, that the actual local IFN-γ concentrations in an *in vivo* setting are difficult to assess, and the cytokine concentration is subject to degradation, uptake, and receptor binding by target cells. As the infection itself had only little effects on the surface molecule expression, it is assumed that the major phenotypical changes occurring during infection are due to IFN-γ production in the pro-inflammatory setting. Taking these experiments as basis, the infection alone would go almost unnoticed regarding the expression of HLA-ABC, HLA-DR, or ICAM-1. However, IFN-γ, but not the *T. cruzi* infection per se, induce an upregulation of HLA-ABC and HLA-DR. Therefore, IFN-γ stimulated keratinocytes might act as antigen-presenting cells for CD8^+^ and CD4^+^ T cells. A similar scenario is also described in previous studies [31,32]. We also observe an upregulation of ICAM-1, an adhesion molecule that is upregulated at sites of local inflammation that mediates the recruitment of leukocytes and has a direct stimulatory effect on NK cells and CD8^+^ T cells [33,34]. Black et al. have demonstrated an upregulation of ICAM-1 expression by keratinocytes through IFN-γ stimulation, which is in line with our results [31]. Importantly, MIC-A/B was upregulated in infected as well as IFN-γ stimulated cells. MIC-A/B is an MHC class I related protein and stimulates CD8^+^ T cells and NK cells via the receptor NKG2D [35].

Of particular interest are the expression of the inhibitory receptors PD-L1 and PD-L2, since both can inhibit immune cell activation by binding to PD-1, which is expressed on T and NK cells [36–38]. Both, PD-L1 and PD-L2 are upregulated in *T. cruzi* infected and IFN-γ stimulated keratinocytes. While an interaction of PD-L1 and/or PD-L2 with PD-1 is typically associated with a reduced immune response, it might also be a counter regulatory mechanism for limiting tissue damage in an infectious setting [39]. This is in accordance with the finding that a blockade of PD-1/PD-L1 interaction was associated with increased heart pathology in murine models of acute and chronic *T. cruzi* infection [40,41]. On the other hand, PD-L1/-L2 expression could impede parasite clearance by preventing an effective immune response. For instance, Gutierrez et al. observed a reduced parasite load and augmented inflammatory response upon PD-1/PD-L1 blockade [41]. HVEM is another receptor that was induced upon IFN-γ stimulation. Depending on the ligand, HVEM can mediate either activating or inhibitory signals. Ligation to CD160 on T cells, for example, has an inhibitory effect, whereas binding to CD160 on a NK cell leads to their activation [42,43]. An increased surface expression of CD160 on CD4^+^ and CD8^+^ T cells has been found in acutely and chronically *T. cruzi* infected mice [44]. Other inhibitory receptors expressed by CD8^+^ T cells and NK cells are Tim-3 and TIGIT. Their respective ligands, Galectin-9 and CD155, were found to be upregulated by keratinocytes upon IFN-γ stimulation in this study. To our knowledge, induction of these molecules on keratinocytes has not been addressed by any studies so far. It is intriguing to speculate that these might serve as potential counter-regulatory mechanism to prevent inflammation of the skin.

Since we observed the induction of inhibitory and activating ligands, it is not clear which effect dominates. However, since we observed a strong activation of NK cells and an induction of their antimicrobial activity to infected keratinocytes at least for NK cells activating pathways are dominating. This may have the potential to strongly reduce the parasite inoculum during the initial infection, which might have a profound effect on the later course of infection.

In conclusion, our study demonstrates that *T. cruzi* has the capability to infect hPEK. IFN-γ, known to be evaluated during the acute and the chronic stage of disease, resulted in the induction of a plethora of immunomodulatory ligands on the surface of these crucial epithelial cells. Keratinocytes, are able to effectively activate NK cells, leading to IFN-γ production and secretion of cytotoxicity-mediating effector molecules. This underscores the importance of NK cells as primary responders in skin immunity. Additionally, NK cell-mediated lysis of *T. cruzi* infected keratinocytes was confirmed using the Opera Phenix system. Our findings suggest that NK cells play a critical role in parasite control during the acute phase of infection in the skin, potentially reducing the residual parasitic load. Further investigations are needed to identify specific mechanisms of signaling between keratinocytes and NK cells and to explore whether redundant systems, such as soluble mediators (e.g., soluble ligand/receptors) contribute to NK cell activation. In addition, synergistic interactions of NK cells with other innate immune cells like macrophages and/or neutrophils which are also abundant in the skin after mechanical injury might leverage trypanocidal activity.

## Materials and methods

### Ethics statement

Keratinocytes were isolated from human 6 mm biopsies kindly provided by healthy donors. Donors gave written informed consent before the donation of skin and matching blood samples. The project was approved by the ethics committee of the Elbe Kliniken Stade-Buxtehude.

### Isolation of Peripheral Blood Mononuclear Cells

Blood was drawn from the median cubital vein using sterile Lithium-Heparin S-Monovetten (Sarsted) and stored for a maximum of 8h at RT. Peripheral Blood Mononuclear Cells (PBMCs) were isolated from whole blood of healthy volunteers using SepMate tubes (StemCell technologies) as described by Mackroth et al. [45]. PBMCs were cryopreserved or used for co-cultivation experiments.

### Isolation of Primary Keratinocytes

Keratinocytes were isolated from human 6 mm biopsies kindly provided by healthy donors. The age and sex of the donors, as well as the biopsy site, are listed in Table 1. Biopsies were stored at 4°C in a physiologic solution for a maximum of 48 h. Under sterile conditions, primary keratinocytes were isolated as described in [46]. Briefly, hair and fatty tissue were removed, and tissue was washed with Dulbecco’s PBS (1x), without Ca & Mg, without Phenol Red (Capricorn scientific), rinsed in 70% ethanol for 10 sec, and washed again in PBS. The skin was cut into 2–3 mm thin stripes and incubated overnight at 4°C in Dispase II (Sigma-Aldrich). On the next day, the epidermis was separated from the dermis and collected in PBS. Single cells were obtained by incubating the epidermis in 1 ml of trypsin-EDTA solution (PAA Laboratories) for 5 min at 37°C, followed by mechanical segregation. Digestion was stopped and after 5 min centrifugation at 200 g, the supernatant was aspirated and cells were resuspended in keratinocyte growth medium (KGM). Cells were cultivated in T25 cell flasks or stored in cryovials in liquid nitrogen at -196°C.

**Table 1:** Information about the donors.

Primary Keratinocytes ID	Age (years)	Sex	Biopsy site
PK1	39	female	upper arm
PK2	54	male	upper arm
PK6	55	female	inguinal region

### Cultivation of Keratinocytes

Keratinocytes were cultivated in keratinocyte growth medium (KGM; DermaLife K medium complete kit (Lifeline Cell Technology), 100 U/ml Penicillin, 100 U/ml Streptomycin (Capricorn Scientific) in T25 cell culture flasks at a density of 0.2–0.5 x 10^6^ cells per flask or in T75 cell culture flasks at a density of 0.5–1.10^6^ cells per flask. For cultivation, 1 μg/ml of phenol red (Sigma-Aldrich) was added to the medium. The flasks were incubated at 37°C and 5% CO_2_ atmosphere. Cells were passaged at a confluency of > 80% and the medium was changed in case confluency was not reached after 3–4 days. Primary keratinocytes were passaged by washing the cell layer with PBS, followed by 10–15 min incubation with Trypsin-0.1% EDTA (Capricorn Scientific) at 37°C. The keratinocytes were used for assays at passages 3–4. Keratinocytes were tested negative for mycoplasma.

### Cryopreservation

Keratinocytes were cryopreserved by resuspending 0.5–1 x 10^6^ cells per 1 ml of freezing medium. PBMCs were frozen at 1 x 10^7^ cells per 1 ml of freezing medium (FCS, 10% DMSO (Sigma-Aldrich)). Keratinocytes were thawed by resuspension in pre-warmed washing medium (DMEM (PAN Biotech), 10% FCS, 100 U/ml Penicillin, 100 U/ml Streptomycin, 2 mM L-Glutamine (Capricorn Scientific)).

### Parasite Culture

*T. cruzi* Brazil strain DTU I [47] was kindly provided from Instituto Nacional de Parasitología Dr. Mario Fatala Chaben Buenos Aires, Argentinien. All work involving viable *T. cruzi* parasites was conducted under biosafety level 3 conditions (BSL-3). Glioblastoma 86HG39 cells were used to keep parasites alive *in vitro* and *T. cruzi* parasites were passages as described in [11].

### Infection of Keratinocytes

Keratinocytes were seeded one day before infection. The number of cells that were seeded per well were optimized for confluency of 60% 24 h after seeding. In 24-well plates 4x10^4^, 48-well plates 25x10^4^ and in 96-well plates 6.5 x10^3^ keratinocytes per well. Additional wells were seeded per plate for counting of cells before infection, as well as before the addition of NK cells. Before *T. cruzi* parasites were added, the medium was changed. Non-infected controls were included in all experiments. The infection with *T. cruzi* parasites was performed as described in [11]. The parasites were resuspended in an appropriate volume of pre-warmed KGM. The MOI of 1:1, 3:1, or 6:1 was used to determine the infection rates of *T. cruzi* Brazil and *T. cruzi* Tulahuen in primary keratinocytes. For all other experiments, an MOI of 3:1 was used. The keratinocytes were co-cultivated with the parasites in KGM for 24 h at 37°C and 5% CO_2_ to allow parasite entry. 24 h after infection, the wells were washed once with medium to remove free parasites, and fresh medium was added.

### Immunofluorescence Staining

Keratinocytes were seeded on chamber slides and infected with *T. cruzi* 24 h later. Every 24 h up to 96 h post-infection (p.i.), chamber slides were washed twice with PBS and the cells fixed with 4% PFA for 45 min at RT. The chamber slides were stored in 200 μl of PBS per well at 4°C. For staining, the cells were permeabilized with 0.1% Triton-X-100 (Sigma-Aldrich) in PBS for 5 min, followed by three washing steps with PBS. Cells were incubated with 100 μl of rabbit anti-*T. cruzi* serum (1:2000) and anti-cytokeratin pan antibody (1:100, Scientific) in PBS for 1.5 h at 37°C. Following three washing steps with PBS, the cells were incubated with 100 μl of secondary antibody mix for 45 min at RT protected from light. The secondary antibody mix contained Alexa Fluor 568 conjugated goat anti-rabbit IgG antibody (1:200) and Alexa Fluor 488 conjugated goat anti-mouse IgG antibody (1:200, both from LifeTechnologies) in PBS. The wells were washed PBS, after which the chambers were removed from the slide. One drop of Roti -Mount FluorCare DAPI (Carl Roth) was added per well and a coverslip was applied. The slides were imaged using a Zeiss Axio Imager M.1 fluorescence microscope. Image overlays were created using the FIJI ImageJ software.

### Immunofluorescence Staining for High Content Screening

The High Content Screening (HCS) system Opera Phenix and Harmony software (version 4.6 from PerkinElmer) were used to determine the total number of cells, the infection rate and the number of trypanosomes in the infected keratinocytes. Cells were seeded in black 96-well plates (CellCarrier) plates and infected with *T. cruzi* Brazil or *T. cruzi* Tulahuen 24 h later. At different time points, the wells were carefully washed twice with pre-warmed PBS and fixed for 45 min with 4% PFA at RT for analysis. After washing with PBS, the plates were stored at 4°C in 200 μl of PBS. For immunofluorescence staining, the wells were washed twice with HCS wash buffer (0.1% Triton-X-100 (Sigma-Aldrich) in PBS for 5 min. All washing and incubation steps were performed under gentle shaking at 300 rpm and RT. The cells were then permeabilized with HCS permeabilization buffer (PBS, 0.1% Triton-X-100, 50 mM NH_4_Cl (Carl Roth)) for 15 min, followed by 30 min blocking with HCS blocking solution (PBS, 0.1% Triton X-100, 2% BSA (SERVA) *T. cruzi* was stained by adding mouse anti-*T. cruzi* serum diluted 1:2000 in blocking solution for 90 min. After three washing steps with wash buffer for 5 min each, 60 μl of the secondary antibody mixture were added and incubated for 60 min protected from light. As a secondary antibody, anti-mouse IgG AF647 (Life Technologies) was used at 1:8000 dilution in blocking solution together with 0.01 mg/ml DAPI (Thermo Fisher Scientific). The wells were washed with wash buffer and PBS. The plate was stored in 200 μl of PBS at 4°C protected from light until it was measured at the Opera Phenix.

The Opera Phenix is a confocal imaging system that automatically creates several images per well. Each well is divided into an 11x11 grid, from which 15 sections were selected for image acquisition. The images can be analyzed with the Harmony software and a custom-made image analysis sequence. Here, the output generated by the analysis sequence included the number of cells, percentage of infected cells, number of trypanosomes, and trypanosomes per infected cell. Cells were detected via the nucleus and a weak cytoplasmic background signal that originates from the unspecific binding of the primary antibody. The *T. cruzi* parasites were detected via a strong AF647 signal together with a small DAPI stained spot reflecting the parasitic nucleus. Several parameters were included to reliably detect *T. cruzi* parasites and the full analysis sequence can be found in Table 2.

**Table 2:** Full analysis sequence Opera Phenix confocal imaging system. The customed analytical sequence was developed employing Harmony software version 4.6 and can be employed for images produced using the Opera Phenix confocal imaging system.

**Input Image**	**Input**		
	**Flatfield Correction:** None		
	Brightfield Correction		
	**Stack processing:** Maximum Projection		
	**Min. Global Binning:** Dynamic		
**Find Nuclei**	**Input**	**Method**	**Output**
	**Channel:** DAPI	**Method:** C	Output Population: cells
	**ROI:** None	Common Threshold: 0.02	
		Area: > 80 μm^2^	
		Split Factor: 16.9	
		Individual Threshold: 0.14	
		Contrast: > -0.71	
**Find Cytoplasm**	**Input**	**Method**	**Output**
	**Channel:** Alexa 647	**Method:** D	
	**Nuclei:** cells	Individual Threshold: 0.06	
**Calculate Intensity Properties**	**Input**	**Method**	**Output**
	**Channel:** DAPI	**Method:** Standard	Property Prefix: Intensity Nucleus DAPI
	**Population:** cells	Mean	
	**Region:** Nucleus		
**Calculate Intensity Properties (2)**	**Input**	**Method**	**Output**
	**Channel:** Alexa 647	**Method:** Standard	Property Prefix: Intensity Cytoplasm Alexa 647
	**Population:** cells	Mean	
	**Region:** Cytoplasm		
**Calculate Morphology Properties**	**Input**	**Method**	**Output**
	**Population:** cells	**Method:** Standard	Property Prefix: Cell
	**Region:** Cell	Area	
		Roundness	
**Find Spots**	**Input**	**Method**	**Output**
	**Channel:** Alexa 647	**Method:** B	Output Population: Spots
	**ROI:** cells	Detection Sensitivity: 0.14	
	**ROI Region:** Cell	Splitting Coefficient: 0.889	
		Calculate Spot Properties	
**Calculate Morphology Properties (3)**	**Input**	**Method**	**Output**
	**Population:** Spots	**Method:** Standard	Property Prefix: Spot
	**Region:** Spot	Area	
		Roundness	
**Calculate Intensity Properties (3)**	**Input**	**Method**	**Output**
	**Channel:** Alexa 647	**Method:** Standard	Property Prefix: Intensity Spot Alexa 647
	**Population:** Spots	Mean	
	**Region:** Spot		
**Calculate Intensity Properties (4)**	**Input**	**Method**	**Output**
	**Channel:** DAPI	**Method:** Standard	Property Prefix: Intensity Spot DAPI
	**Population:** Spots	Mean	
	**Region:** Spot		
**Calculate Intensity Properties (5)**	**Input**	**Method**	**Output**
	**Channel:** Alexa 647	**Method:** Standard	Property Prefix: Intensity Cytoplasm Alexa 647
	**Population:** cells	Mean	
	**Region:** Cytoplasm		
**Calculate Properties (2)**	**Input**	**Method**	**Output**
	**Population:** Spots	**Method:** By Formula	Output Property: Alexa/DAPI Ratio
		Formula: A/B	
		Variable A: Intensity Spot Alexa 647 Mean	
		Variable B: Intensity Spot DAPI Mean	
**Select Population**	**Input**	**Method**	**Output**
	**Population:** Spots	**Method:** Filter by Property	Output Population: interesting Spots
		Spot Roundness: < = 1.2	
		Spot Area [px^2^]: < = 800	
		Spot Area [px^2^]: > = 46	
		Spot Roundness: > 0.25	
		Relative Spot Intensity: > 0.4	
		Spot Contrast: > 0.1	
		Region Intensity: > 200	
		Corrected Spot Intensity: > 1400	
		Intensity Spot DAPI Mean: > 400	
		Uncorrected Spot Peak Intensity: > 2500	
		Boolean Operations: F1 and F2 and F3 and F4 and F5 and F6 and F7 and F8 and F9 and F10	
**Select Population (2)**	**Input**	**Method**	**Output**
	**Population:** interesting Spots	**Method:** Linear Classifier	Output Population A: Trypanosoma selected
		Number of Classes: 2	Output Popualtion B: false-positive
		Relative Spot Intensity	
		Corrected Spot Intensity	
		Uncorrected Spot Peak Intensity	
		Spot Contrast	
		Spot Background Intensity	
		Spot Area [px^2^]	
		Region Intensity	
		Spot to Region Intensity	
		Spot Area [μm^2^]	
		Spot Roundness	
		Intensity Spot Alexa 568 Mean	
		Intensity Spot DAPI Mean	
**Select Population (6)**	**Input**	**Method**	**Output**
	**Population:** interesting Spots	**Method:** Filter by Property	Output Population: Trypanosoma
		Regression A-B: < 15.1	
**Calculate Properties**	**Input**	**Method**	**Output**
	**Population:** cells	**Method:** By Related Population	Property Suffix: per Cell
		Related Population: Trypanosoma	
		Number of Trypanosoma	
**Select Population (4)**	**Input**	**Method**	**Output**
	**Population:** cells	**Method:** Filter by Property	Output Population: inf. cells
		Number of Trypanosoma- per Cell: > 0	
**Select Population (7)**	**Input**	**Method**	**Output**
	**Population:** cells	**Method:** Filter by Property	Output Population: double inf.cells
		Number of Trypanosoma- per Cell: > 2	
**Select Population (5)**	**Input**	**Method**	**Output**
	**Population:** inf. cells	**Method:** Filter by Property	Output Population: massive inf. Cells
		Number of Trypanosoma- per Cell: > 3	
**Define Results**	**Results**		
	**Method:** List of Outputs		
	**Population: inf. cells**		
	Number of Objects		
	**Population: cells**		
	Number of Objects		
	**Population: Trypanosoma**		
	Number of Objects		
	**Population: double inf.cells**		
	Number of Objects		
	**Population: masive inf. cells**		
	Number of Objects		
	**Method:** Formula Output		
	Formula: a/b		
	Population Type: Objects		
	Variable A: Trypanosoma—Number of Objects		
	Variable B: cells—Number of Objects		
	Output Name: Trypanosoma per cell		
	**Method:** Formula Output		
	Formula: a/b		
	Population Type: Objects		
	Variable A: Trypanosoma—Number of Objects		
	Variable B: inf. cells—Number of Objects		
	Output Name: Trypanosoma per inf. cell		
	**Method:** Formula Output		
	Formula: a/b*100		
	Population Type: Objects		
	Variable A: inf. cells—Number of Objects		
	Variable B: cells—Number of Objects		
	Output Name: perc.inf.cells		
	**Object Results**		
	Population: inf. cells: Use Selected Well Results		
	Population: cells: Use Selected Well Results		
	Population: Spots: None		
	Population: interesting Spots: None		
	Population: Trypanosoma selected: None		
	Population: false-positive: None		
	Population: Trypanosoma: Use Selected Well Results		
	Population: double inf.cells: Use Selected Well Results		
	Population: massive inf. cells: Use Selected Well Results		

### Stimulation of Primary Keratinocytes

Primary Keratinocytes from three donors were seeded in 24-well plates and stimulated with KGM Media supplemented either with 0.1 ng/ml or 10 ng/ml of recombinant human IFN-γ (PeproTech), or infected with *T. cruzi* Brazil. The keratinocytes were stained for various surface markers using flow cytometry. The MOI used was 3:1. Furthermore, if cell counts allowed it negative control per donor were included as neither infected nor stimulated. The plates were incubated for 72 h at 37°C and 5% CO_2_. 24 h p.i., the medium from all wells was removed, the wells were washed once with KGM, and fresh KGM was added. 72 h p.i., the keratinocytes were washed with cold PBS and detached by 15 min incubation at 37°C with 100 μl/well of trypsin (TrypLE, Life Technologies). The reaction was stopped by adding 150 μl of DMEM supplemented with 10% FCS, after which the cells were thoroughly resuspended and transferred to a 96-well U-bottom plate for flow cytometry staining.

### Isolation of NK cells and Co-Culture Experiments

NK cells were isolated from frozen PBMCs by negative selection using the MojoSort Human NK Cell Isolation Kit (BioLegend) following the manufacturer’s instructions. The NK cells were counted and were resuspended in cRPMI supplemented with 5 ng/ml rhIL-15 (PeproTech) and rested overnight in a 96-well U-bottom plate at 37°C and 5% CO_2_. Subsequently, NK cells were incubated with *T. cruzi* Brazil infected and non-infected primary keratinocytes. The keratinocytes were washed once with cRPMI, and the NK cells were added to cRMPI supplemented with 1 ng/ml rhIL-15. For flow cytometric analysis, the NK cells were added at an E: T ratio of 1:10. For supernatant and HCS analysis, E:T ratios of 1:3 and 10:1 were used, respectively. After the NK cells were added, the plates were centrifuged at 100 x g for 30 sec to accelerate the contact between the target and effector cells. The NK cells were co-cultured with the keratinocytes for 24 h. For the last 5 h of co-culture, Brefeldin A (Biolegend) and APC-Cy7 conjugated anti-CD107a antibody (1:200) were added to the wells for flow cytometry staining. The plate was centrifuged for 30 sec at 100 x g to re-establish cell contact. After 24 h of co-culture, the NK cells were harvested and analyzed using flow cytometry. The co-culture supernatants were collected and analyzed using a multiplex bead assay, Briefly, the NK cells in the 24-well plate were harvested by pipetting the culture supernatant up and down and transferring it to a 96-well U-bottom plate for flow cytometry staining. For cell culture supernatants, plate was centrifuged for 5 min at 500 g and 4°C, after which the supernatants were carefully transferred to a 96-well U-bottom plate. The plate was centrifuged for 15 min at 2671 g and 4°C to pelletize any parasites, and the supernatant was stored at -20°C. For HCS analysis, the CellCarrier plate was washed and fixed as described above.

### Flow Cytometry Staining

The flow cytometry staining was performed as described in [11]. Briefly, all washing steps were performed at 4°C for 5 min at 400 g (keratinocytes) or 500 g (NK cells). For each staining, a negative control, as well as an unstained control were performed. First, a LIVE/DEAD fixable blue stain (Thermo fisher) was performed. Subsequently, cells were washed with FACS buffer (PBS, 2% FCS, 2 mM EDTA), surface staining antibodies were added and incubated for 30 minutes at 4°C. For intracellular cytokine staining, cells were fixed and permeabilized using the Foxp3/Transcription Factor Buffer Set (eBioscience) for 45 minutes at RT. Following fixation and permeabilization, intracellular staining was performed for 30 minutes at 4°C. Measurements were taken using Cytek Aurora spectral flow cytometer and SpectroFlo software. The data was analyzed using FlowJo. Gates were established based on fluorescence minus one (FMO) controls. The gating strategy is depicted in Fig C and Fig G in S1 Appendix. Table 3 provides a list of antibodies used in flow cytometric analysis.

**Table 3:** List of Antibodies.

Target	Fluorophore	Clone	Dilution	Company
CD107a (LAMP-1)	APC-Cy7	H4A3	1:200	BioLegend, Inc., San Diego, USA
CD155 (PVR)	PE/Dazzle 594	SKII.4	1:100	BioLegend, Inc., San Diego, USA
CD16	PE/Dazzle 594	3G8	1:100	BioLegend, Inc., San Diego, USA
CD270 (HVEM)	PE	122	1:100	BioLegend, Inc., San Diego, USA
CD273 (B7-DC, PD-L2)	PE	24F.10C12	1:400	BioLegend, Inc., San Diego, USA
CD274 (B7-H1, PD-L1)	BV421	29E.2A3	1:100	BioLegend, Inc., San Diego, USA
CD3	BUV737	SK7	1:200	BD Biosciences, New Jersey, USA
CD54 (ICAM-1)	Alexa Fluor 700	HA58	1:200	BioLegend, Inc., San Diego, USA
CD56	BUV395	NCAM16.2	1:100	BD Biosciences, New Jersey, USA
Galectin-9	PE-Cy7	9M1-3	1:200	BioLegend, Inc., San Diego, USA
HLA-A,B,C	FITC	W6/32	1:200	BioLegend, Inc., San Diego, USA
HLA-Bw4	APC	REA274	1:100	Miltenyi Biotec B.V. & Co. KG, Bergisch Gladbach, Germany
HLA-C	BUV395	DT-9	1:100	BD Biosciences, New Jersey, USA
HLA-DR	BV650	L243	1:100	BioLegend, Inc., San Diego, USA
HLA-E	FITC	REA1031	1:100	Miltenyi Biotec B.V. & Co. KG, Bergisch Gladbach, Germany
HLA-F	APC	3D11/HLA-F	1:50	BioLegend, Inc., San Diego, USA
HLA-G	PE/Dazzle 594	87G	1:50	BioLegend, Inc., San Diego, USA
MIC-A/MIC-B	PE-Cy7	6D4	1:50	BioLegend, Inc., San Diego, USA

### Cytokine Measurement

Cytokines were quantified using the LEGENDplex Human CD8/NK Panel 13-plex (Cat.No #740267 Lot. B330268) from Biolegend. This is a bead-based immunoassay able to quantify the concentration of different analytes in co-culture supernatants. The assay was performed according to the manufacturer’s instructions and measured using at the BD Accuri C6 flow cytometer. The data was analyzed using the LEGENDPlex Data Analysis Software Suite.

### Statistics

Statistical analysis was performed using GraphPad Prism 9. The data were tested for normal distribution using a Shapiro-Wilk normality test. An unpaired t-test with Welch’s correction was performed to evaluate the differences between the two groups. In case the data did not pass the normality test, a Mann-Whitney test was performed. For analysis of more than two groups, RM one-way ANOVA and Holm-Sidak’s multiple comparison test for values that follow normal distribution and Friedman test and Dunn’s multiple comparison test for values that do not. Results were rated as statistically significant when * p ≤ 0.05; ** p ≤ 0.01; *** p ≤ 0.001; **** p ≤ 0.0001.

## Supporting information

S1 Appendix Fig A Infection of human primary keratinocytes with *T*. *cruzi* Tulahuen. Primary keratinocytes were infected with *T*. *cruzi* Tulahuen at a MOI of 3:1 for 24 h, 48 h, and 96 h. Keratinocytes (green) and trypanosomes (red) were visualized by indirect immunofluorescence using a pan anti-cytokeratin antibody, polyclonal anti-*T*. *cruzi* serum, and DAPI. Images were obtained at 200x magnification. n.i., not infected; TcT inf., *T*. *cruzi* Tulahuen infected. Fig B Scheme of autologous co-cultivation. Figure created with a licensed version of Biorender. 1. Seeding and cultivate keratinocytes to get confluency. 2. Infect keratinocytes with cell culture derived *T. cruzi* trypomastigotes, wash trypomastigotes that were not successful in invading cells after 24 h. 3. After 72 h amastigotes are already visible and have filled up the cells but are not yet transforming into the blood trypomastigotes. 4. Collect fresh blood donation from same donor as the keratinocytes and perform magnetic sorting from NK cells. 5. Add NK cells in different ratios. 6. Co-cultivate autologous NK cells and *T. cruzi*-infected keratinocytes for 24h. 7. Collect supernatant from the co-culture to measure soluble mediators in multiplex cytokine bead-based immunoassay. 8. Collect not adherent NK cells for flow cytometric analysis and determinate phenotype, degranulation status and activation status. 9. Stain cells for automated analysis in Opera Phenix HCS system and analyze the trypanocidal effect of NK cells on *T*. *cruzi*-infected keratinocytes. Fig C Gating strategy for NK cells in the autologous co-culture with Keratinocytes. The gating strategy for analyzing NK cells after being co-cultured with autologous keratinocytes involves defining NK cells based on the expression of CD3^neg^ and CD56^+^. Within the NK cell population, the gating includes CD107a and CD16 (highlighted in blue). The representative gating strategy is shown in the upper panel for co-culture with uninfected keratinocytes and in the middle panel for co-culture with infected keratinocytes. The gates are based on the respective fluorescence minus one (FMO) control, as shown in the lower panel. Additionally, the representative histograms depict the expression of CD107a. Fig D Analysis of cytokines and cytolytic mediators. Release of important NK cell-specific cytolytic mediators in the supernatant of the co-cultures. The fold change was calculated in relation to the non-infected controls. n = 11–12 from 2 donors (PK1, PK2) and 2 experiments each. Data are shown as mean ± SD. Welch’s t tests were performed however, no statistically significant differences were observed in the expression of the molecules analyzed * p ≤ 0.05; ** p ≤ 0.01; *** p ≤ 0.001; **** p ≤ 0.0001; n.i.–non-infected, TcB inf.–*T*. *cruzi* Brazil infected. Fig E Scheme of experimental infection of human primary keratinocytes with *T*. *cruzi* and **IFN-γ**, stimulation. The experimental design involved assessing the surface expression of various molecules in hPEK infected with *T*. *cruzi* or stimulated with IFN-γ using flow cytometry analysis. Untreated hPEK were used as control. hPEK were infected at a MOI of 3:1 or stimulated with 0.1 ng/ml and 10 ng/ml of IFN-γ for 72 h. Figure created with a licensed version of Biorender. Fig F Representative gating strategy of flow cytometric analysis of immune modulatory molecules on the surface of keratinocytes. F1) Representative gating strategy for hPEK based on physical parameters and viability dye. All ligands have been gated on living hPEK. The plots that follow display the representative strategy of various groups, including the unstained and untreated uninfected controls, as well as the TcB infected group treated with either 0.1 ng/ml or 10 ng/ml IFN-γ. F2) Activating ligands HLA-DR, HLA-ABC, ICAM-1 and MIC-A, B. F3)) Inhibitory ligands: PD-L1, PD-L2, HVEM, CD155, Galectin-9. F4) HLA-Molecules: HLA-E, HLA-F, HLA-Bw4+, HLA-C, HLA-G.
(PDF)
