# Supplementary material for: Antimicrobial activity of NK cells to Trypanosoma cruzi infected human primary Keratinocytes
Source: PLoS Negl Trop Dis. 2024 Jul 22;18(7):e0012255. doi: 10.1371/journal.pntd.0012255 (PMC11262665; doi:10.1371/journal.pntd.0012255)

Supplementary Figures – S1 Appendix

Fig A

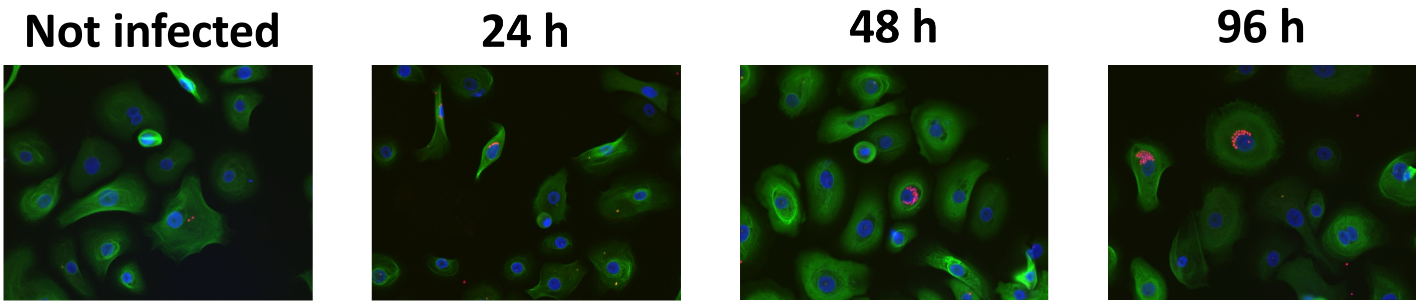

Fig B Schema co-culture experiments

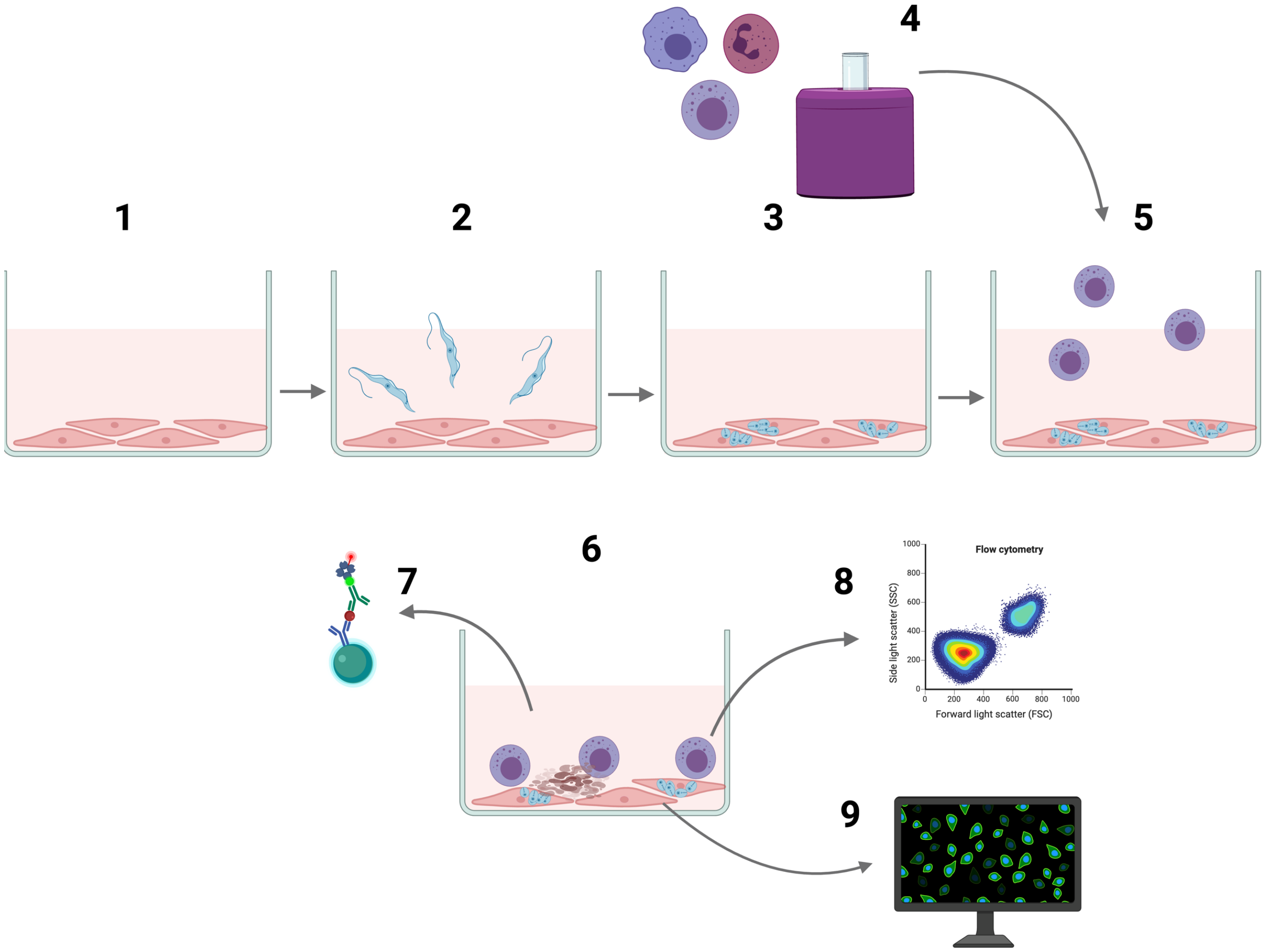

created with Biorender.com

Fig C Gating Strategy and exemplary dot blots co-culture experiments

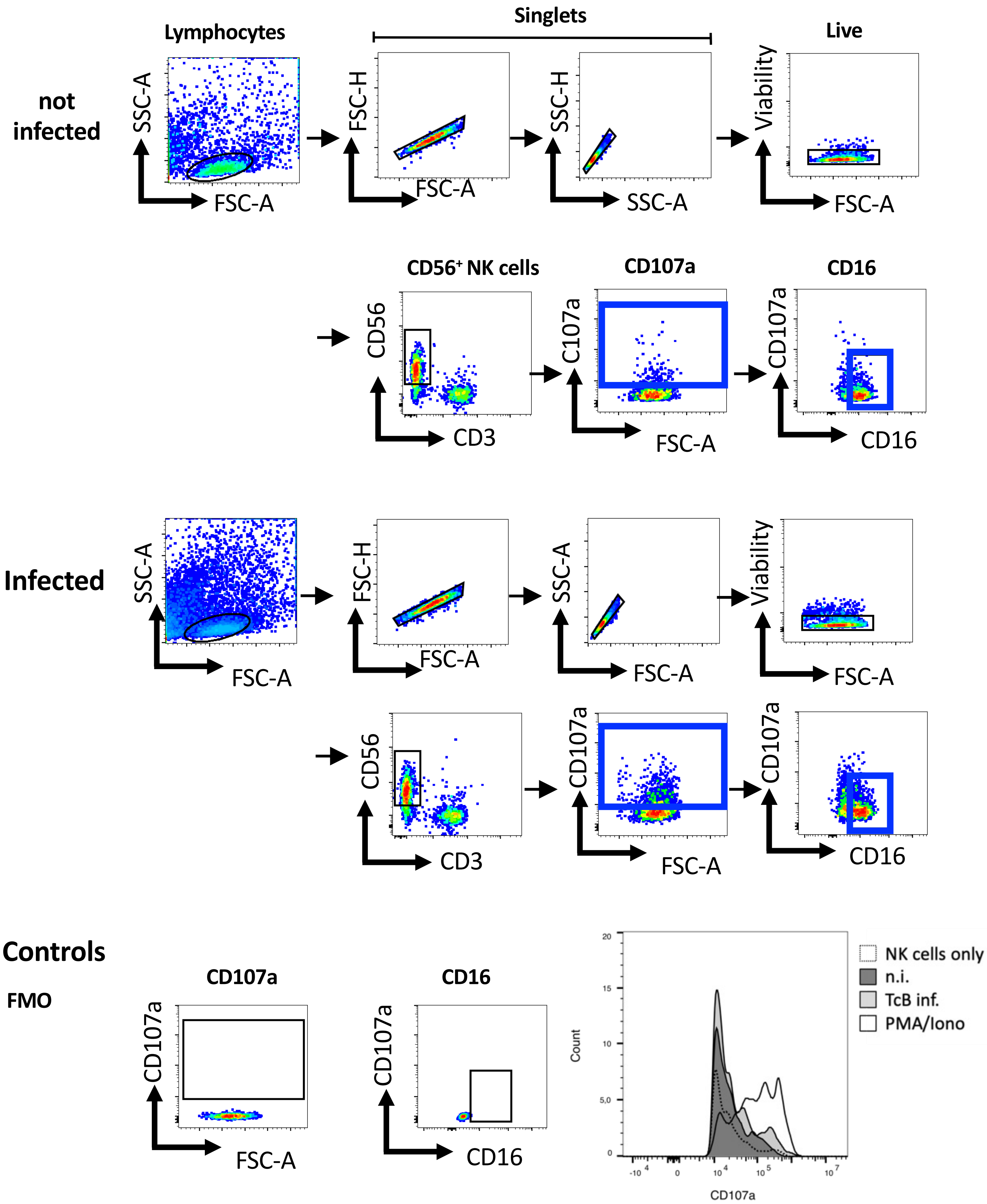

Fig D

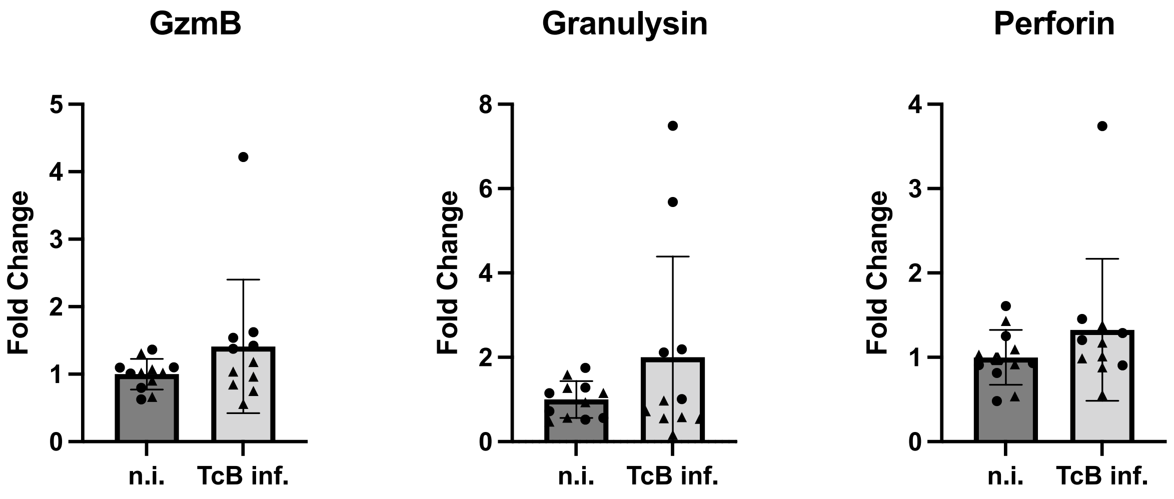

Fig E Scheme human primary keratinocytes of infection T. *cruzi* and IFN-γ stimulation

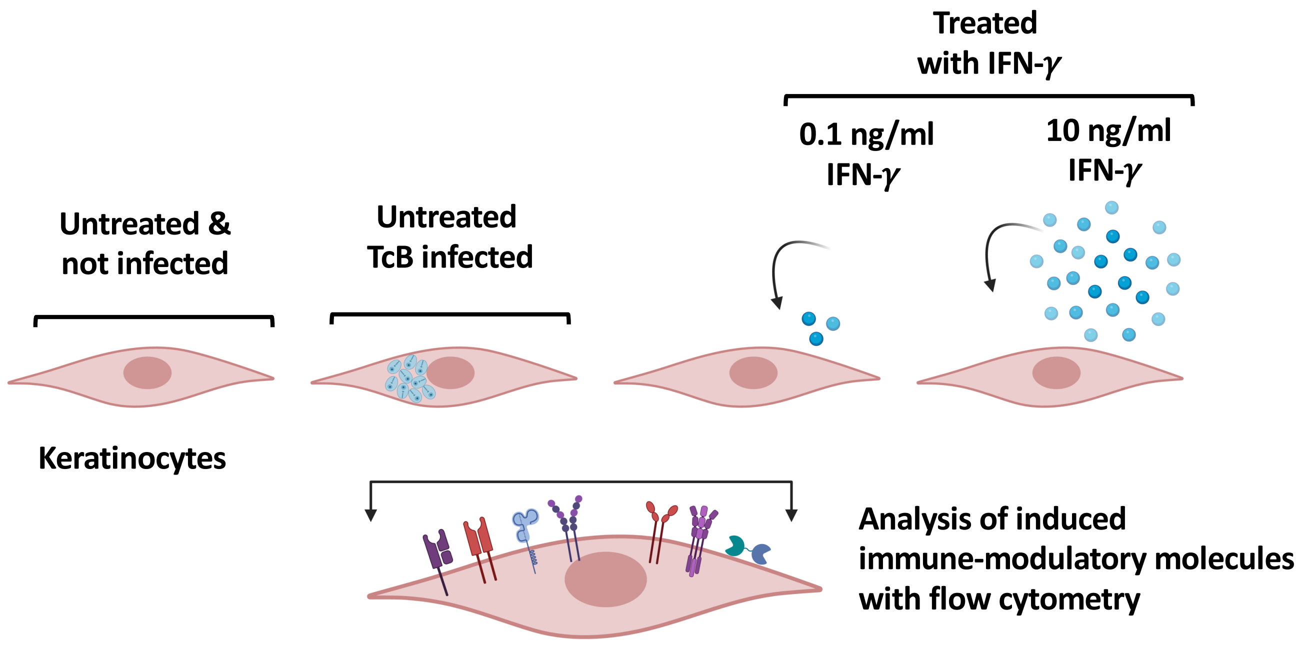

created with Biorender.com

Fig F Gating Strategy and exemplary dot blots

F1

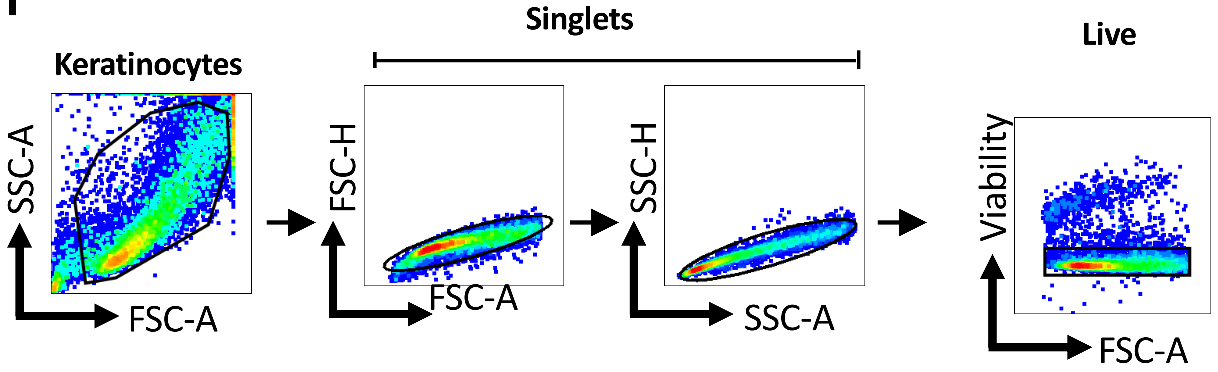

F2 Activating Ligands

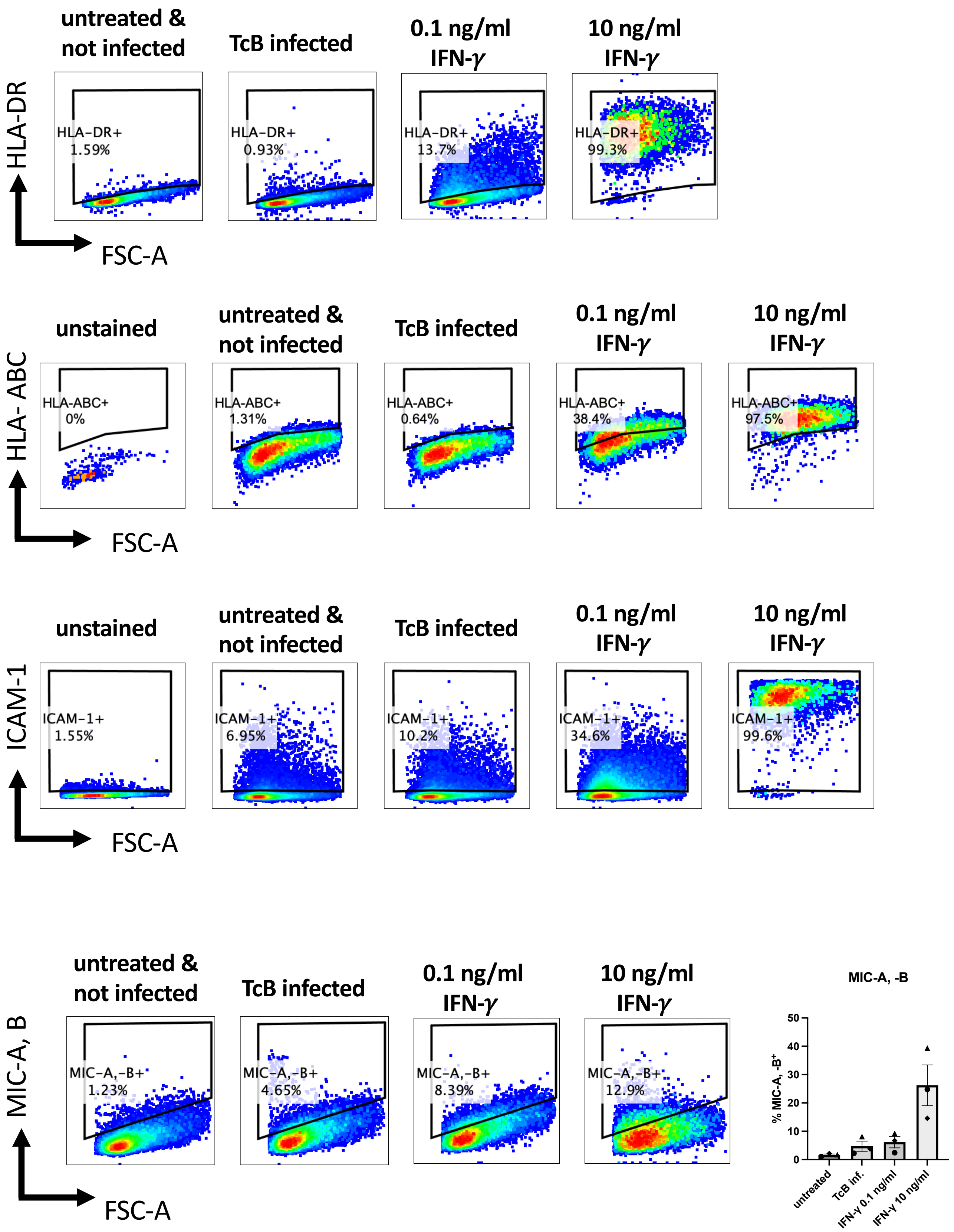

F3 Inhibitory ligands

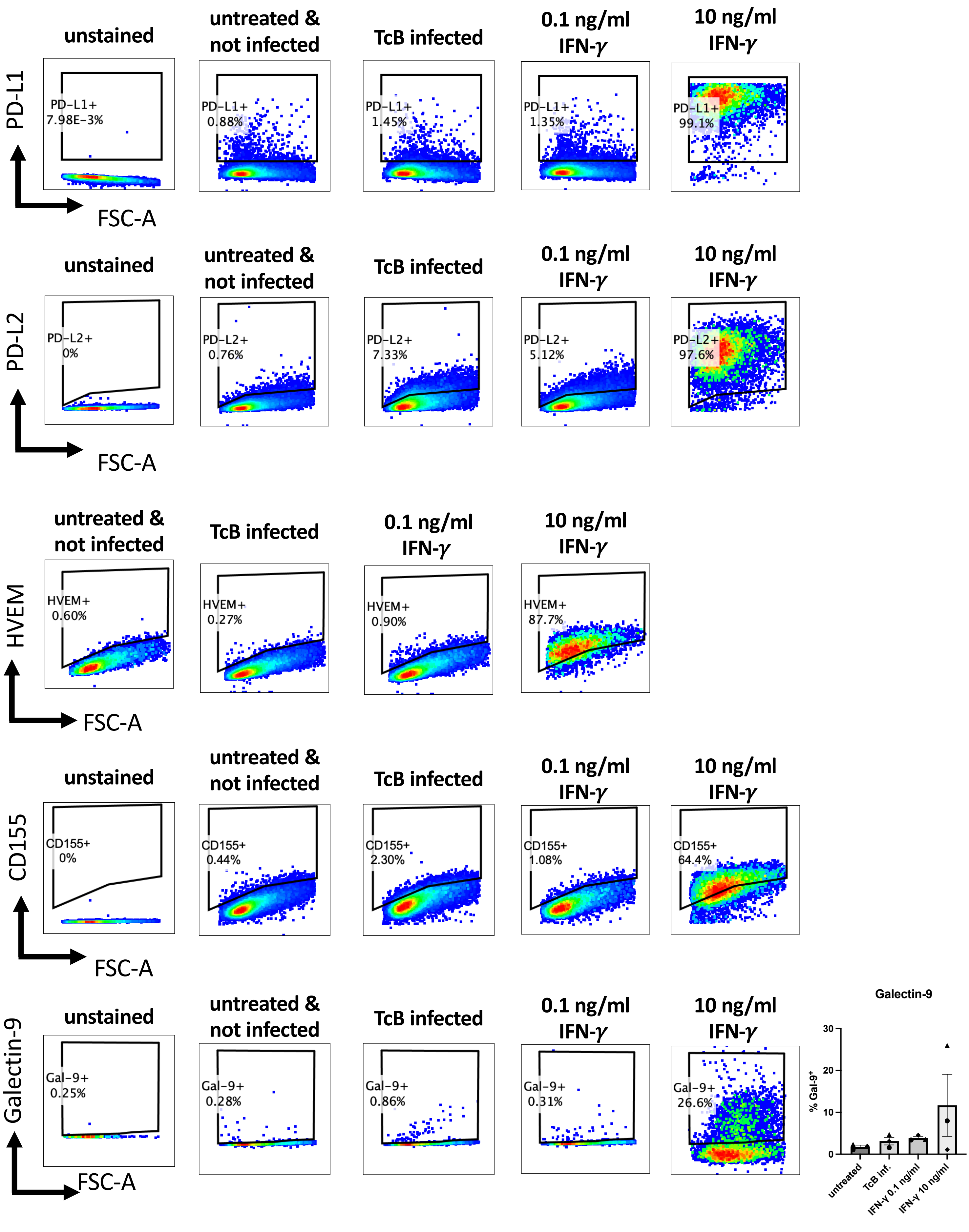

F4 HLA- molecules

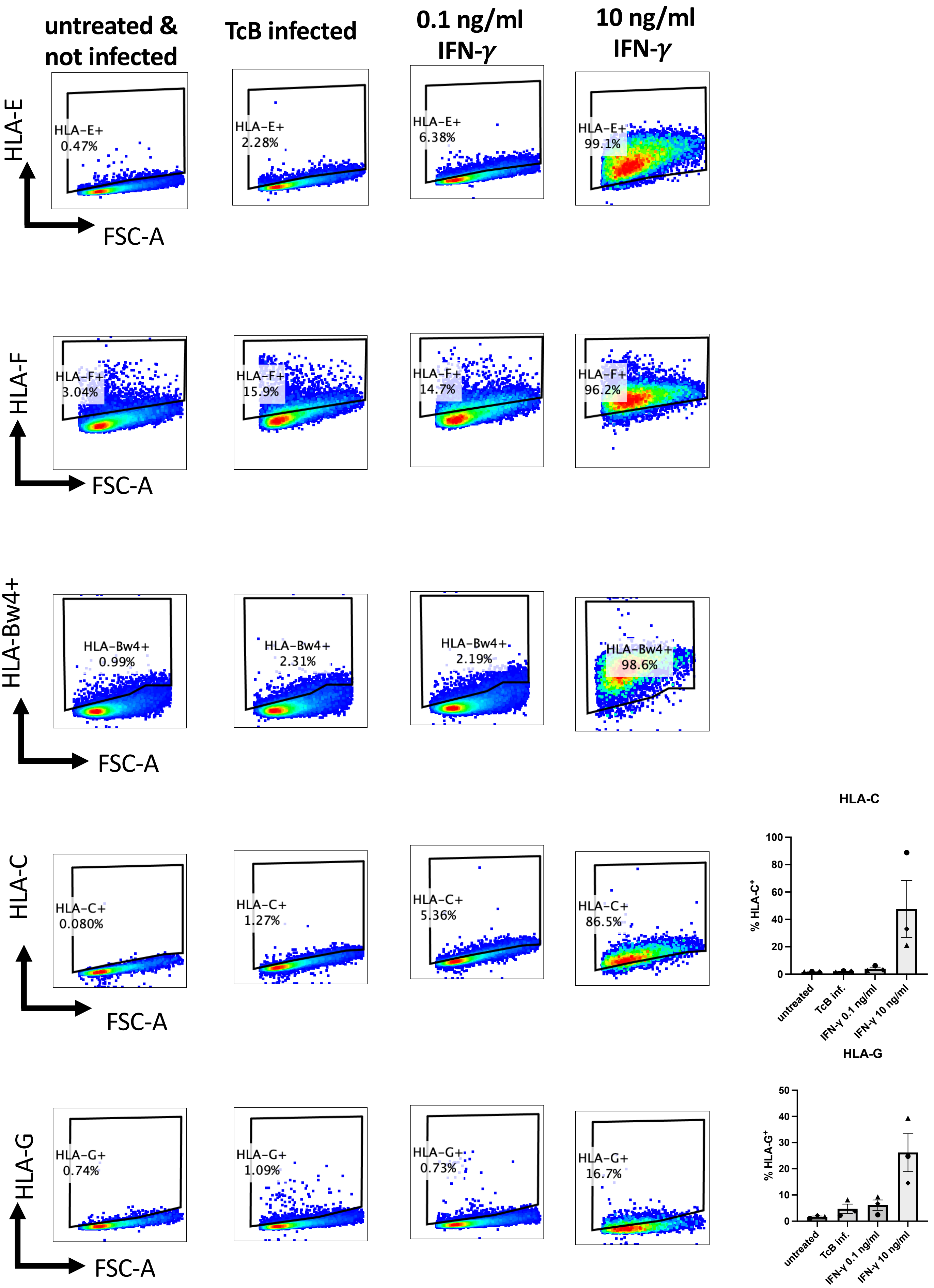

Supplement: S1 Appendix — Fig A Infection of human primary keratinocytes with T. cruzi Tulahuen. Primary keratinocytes were infected with T. cruzi Tulahuen at a MOI of 3:1 for 24 h, 48 h, and 96 h. Keratinocytes (green) and trypanosomes (red) were visualized by indirect immunofluorescence using a pan anti-cytokeratin antibody, polyclonal anti-T. cruzi serum, and DAPI. Images were obtained at 200x magnification. n.i., not infected; TcT inf., T. cruzi Tulahuen infected. Fig B Scheme of autologous co-cultivation. Figure created with a licensed version of Biorender. 1. Seeding and cultivate keratinocytes to get confluency. 2. Infect keratinocytes with cell culture derived T. cruzi trypomastigotes, wash trypomastigotes that were not successful in invading cells after 24 h. 3. After 72 h amastigotes are already visible and have filled up the cells but are not yet transforming into the blood trypomastigotes. 4. Collect fresh blood donation from same donor as the keratinocytes and perform magnetic sorting from NK cells. 5. Add NK cells in different ratios. 6. Co-cultivate autologous NK cells and T. cruzi-infected keratinocytes for 24h. 7. Collect supernatant from the co-culture to measure soluble mediators in multiplex cytokine bead-based immunoassay. 8. Collect not adherent NK cells for flow cytometric analysis and determinate phenotype, degranulation status and activation status. 9. Stain cells for automated analysis in Opera Phenix HCS system and analyze the trypanocidal effect of NK cells on T. cruzi-infected keratinocytes. Fig C Gating strategy for NK cells in the autologous co-culture with Keratinocytes. The gating strategy for analyzing NK cells after being co-cultured with autologous keratinocytes involves defining NK cells based on the expression of CD3neg and CD56+. Within the NK cell population, the gating includes CD107a and CD16 (highlighted in blue). The representative gating strategy is shown in the upper panel for co-culture with uninfected keratinocytes and in the middle panel for c [file pntd.0012255.s001.pdf]
